# Polygenic risk scores in clinical applications – opportunities and challenges

**DOI:** 10.1515/medgen-2026-3016

**Published:** 2026-07-08

**Authors:** Johannes Schumacher, Markus M. Nöthen, Stefanie Heilmann-Heimbach

**Affiliations:** Philipps University Marburg & University Hospital of Marburg Institute of Human Genetics Baldingerstr. 35043 Marburg Germany; University of Bonn & University Hospital of Bonn Institute of Human Genetics Venusberg-Campus 1 53127 Bonn Germany; University Hospital of Bonn Institute of Human Genetics Sigmund-Freud-Str. 25 53105 Bonn Germany

## Abstract

Polygenic risk scores (PRS) are increasingly transitioning from research tools into instruments with direct clinical relevance. This review addresses both opportunities and challenges of PRS across disease contexts. Key applications include risk stratification in multifactorial and monogenic diseases, diagnostic support in etiologically heterogeneous disorders, and pharmacogenomic stratification. These are accompanied by substantial challenges, including limited transferability across diverse populations, insufficient methodological standardization, and barriers to effective risk communication. PRS hold considerable potential to contribute to a more individualized, predictive, and preventive medicine, provided that scientific rigor and responsibility guide their clinical translation.

## Introduction

Polygenic risk scores (PRS) aggregate the effects of multiple common genetic variants across the genome into a single numerical estimate of an individual’s genetic predisposition to a given disease or trait. Over recent years, PRS have evolved from a predominantly research-oriented approach into tools with potential clinical utility across a broad range of possible applications. While early discussions often underestimated their potential, enthusiasm for PRS in clinical practice has increased, albeit outpacing in some instances the available evidence. The aim of the present review is to provide a balanced perspective on the use of PRS in clinical practice. Alongside the opportunities of PRS across different areas of clinical application, we address the specific challenges that must be considered in each case. Rather than focusing on individual diseases, we discuss principles that apply across disease contexts, using specific examples for illustrative purposes only.

## Opportunities for PRS in clinical practice

For clinical implementation, it is essential that individual risk estimates can be translated into clinically actionable measures and meaningful clinical applications, including early detection, preventive strategies, therapeutic interventions, lifestyle modification, and support for diagnostic decision-making. This is essential, since risk estimates are only clinically useful when they can inform decisions on interventions with demonstrated outcome benefits.

The following sections outline key current and emerging clinical applications of PRS, including the identification of individuals at high genetic risk, diagnostic support, and applications in pharmacogenomics. Many of these examples highlight the importance of large population-based biobanks for PRS development and validation, in which individuals undergo long-term longitudinal assessment and from whom information on additional risk factors is collected [1].

### PRS for risk stratification in multifactorial diseases

An established and growing area of research and clinical PRS application is risk stratification at both the individual and the population level. By assigning individuals to a continuous spectrum of genetic risk, those at increased risk of disease can be identified and offered preventive measures. In this context, PRS are particularly informative when integrated into established clinical risk models. Areas in which PRS are expected to play an increasingly important role include cardiovascular and oncological diseases (see the reviews by Li et al. and Tüchler et al. in this issue).

For coronary artery disease (CAD), for example, data from the UK Biobank (UKB) have shown that the incorporation of PRS improves the predictive performance of established risk models [2]. This effect was particularly pronounced in younger individuals (< 50 years). Specifically, individuals with a high PRS who were, according to clinical information (diabetes, hypertension, blood lipids), initially below the threshold for recommending statin therapy (borderline risk group, 5–7.5 % 10-year risk of a cardiovascular event) showed higher rates of cardiovascular events (> 8 %) over 10-year follow-up. Had this elevated cardiovascular event risk been known, these individuals would have met criteria for statin therapy [3]. Within the clinical borderline risk group, this concerned 23 % (1,154 of 4,817 individuals).

In the field of oncology, the recently published WISDOM study from the United States (21,631 participants) provides a compelling example of how PRS can improve risk-adapted breast cancer screening [4]. Integration of PRS into clinical risk models led to changes in screening recommendations for 14 % of women aged 40–49 years and 10 % of women aged 50–74 years. Among women aged 40–49 years, the main change was an earlier start date for screening. Among women aged 50–74 years, the revised recommendations primarily affected those for whom annual mammography had been recommended on the basis of clinical risk models. With the additional use of PRS, 65 % of these women were recommended to undergo biennial mammography, while 21 % were recommended to undergo annual mammography combined with magnetic resonance imaging. These risk-adapted recommendations illustrate the practical potential of PRS-based screening strategies to individualize surveillance programs, by increasing surveillance intensity when genetically warranted and avoiding overscreening when not.

### PRS for risk stratification in monogenic diseases

Despite the large effect sizes of the respective underlying mutations, many monogenic diseases show incomplete penetrance, i.e., only a proportion of mutation carriers develop the disease. In addition to environmental factors, a substantial contributor to this variability is a genetic background that is characterized by polygenic risk. In this context, PRS can make a significant contribution to improving individual disease risk estimates (see the review by Heyne et al. in this issue). Importantly, such refined risk estimates may have direct implications for clinical management, including surveillance intensity, the timing of preventive interventions, and individualized counselling.

In hereditary breast and ovarian cancer (HBOC), the incorporation of PRS into risk prediction is already established. The Breast and Ovarian Analysis of Disease Incidence and Carrier Estimation Algorithm (BOADICEA) model, which is widely used in clinical practice and recommended within several European guidelines, integrates PRS alongside monogenic variant data, family history, and lifestyle factors to generate individualized lifetime risk estimates for breast and ovarian cancer, and represents one of the most advanced examples to date of the integration of PRS into a validated clinical risk tool [5–7]. In this context, a study by Myriad Genetics involving more than 10,000 women with HBOC showed that the impact of PRS is strongly dependent on the causative gene [8]. The modifying effect of PRS was strongest in carriers of mutations in genes with moderate penetrance (*ATM*, *PALB2*, *CHEK2*), and was markedly weaker in carriers of mutations in the highly penetrant genes *BRCA1* and *BRCA2*. Notably, when stratified by PRS percentile, the risk of developing breast cancer by the age of 80 years among *CHEK2* mutation carriers ranged from 6.6 % (lowest 1 %) to 70.6 % (highest 1 %), whereas the variability in risk among *BRCA1* mutation carriers across the same PRS percentiles was lower, ranging from 53.1 % to 91.5 %.

In hereditary nonpolyposis colorectal cancer (HNPCC, Lynch syndrome), which is caused by mutations in the genes *MLH1*, *MSH2*, *MSH6*, or *PMS2*, data from the UKB show that PRS can be used to refine colorectal cancer risk prediction in mutation carriers. The average risk of developing colorectal cancer by the age of 75 years is 35 % [9]. When incorporating PRS, individuals with a low PRS (lowest 20 %) had a colorectal cancer risk of 11.3 %, whereas those with a high PRS (highest 20 %) had a risk of 79.7 %.

### PRS for diagnostic support and differential diagnosis

Beyond risk prediction, PRS also hold promise in terms of facilitating diagnostics, particularly in clinically heterogeneous disorders with overlapping phenotypes. This is especially relevant in psychiatry, in which traditional diagnostic categories are based on symptom clusters, temporal criteria, and functional impairment rather than on underlying biological mechanisms. In such contexts, PRS may help to refine diagnostic classification by capturing differences in genetic liability across related disorders (see the review by Frach et al. in this issue).

For example, the EUropean network of national schizophrenia networks studying Gene-Environment Interactions (EU-GEI) study showed that the combined use of multiple PRS can help distinguish schizophrenia-spectrum disorders from affective disorders (bipolar and major depressive disorder) in individuals with a first episode of psychosis, which is a clinical feature common to both disease groups [10]. Specifically, higher schizophrenia PRS were associated with schizophrenia-spectrum diagnoses, whereas higher PRS for bipolar disorder and major depression were more strongly associated with affective disorders. These findings highlight the potential of PRS to complement clinical assessment and improve diagnostic precision. However, it must be emphasized that PRS do not currently meet the threshold for clinical diagnostic application in psychiatry or other fields, since their discriminative accuracy at the individual level is insufficient, and the findings of the EU-GEI study have not yet been translated into clinical guidelines. The role of PRS in diagnostic classification therefore remains at the investigational stage, and their use in this context should be restricted to research settings until prospective validation data are available. At present, therefore, PRS should be regarded as probabilistic modifiers of disease liability rather than as diagnostic tools.

### Pharmacogenomics: from genetic stratification to PRS

Genetic stratification plays an important role in pharmacogenomic approaches, both in clinical trials and in the prescription and use of approved medications (see the review by Mathey et al. in this issue).

An example of how genetic stratification can be instrumental in securing regulatory approval for a new drug is provided by the Alzheimer’s medication lecanemab, a monoclonal antibody targeting β-amyloid. Following a negative initial regulatory assessment by the European Medicines Agency (EMA) in July 2024, the European Commission granted marketing authorizations in April 2025 for a subsequent conditional approval in Europe in 2025 for a restricted indication, limited to patients with one or no copy of the *APOE4* allele, a population with a more favourable benefit-risk profile due to lower rates of treatment-related adverse effects [11]. This example illustrates how genetic stratification can support the evaluation of benefit-risk balance, thereby enabling the targeted use of therapies in defined patient subgroups despite broader safety concerns.

Turning to PRS specifically, post hoc analyses of several clinical trials, including studies of statin therapy and PCSK9 inhibitors in CAD [12–14], have shown that participants with a high PRS for the respective disease respond better to pharmacological treatments than those with a low PRS [12–15]. However, at the time of writing, the available evidence remains limited and largely exploratory, and these findings should be regarded as hypothesis-generating rather than confirmatory.

Beyond disease-specific PRS, genetic stratification based on PRS tailored directly to pharmacological outcomes, rather than to disease risk, is also considered promising, with respect to both reducing the risk of adverse effects and improving treatment efficacy [16]. A prerequisite for the development of such scores is the availability of large datasets on individual drug response and adverse events. They are expected to offer greater predictive precision for treatment-related outcomes than disease-specific PRS, which were not designed for this purpose.

Ethnic equity is a long-standing concern in pharmacogenomics: non-European populations are markedly underrepresented in pharmacogenomic studies, despite well-documented ancestry-specific differences in the allele frequencies of clinically relevant drug-metabolizing and drug-response variants. For emerging pharmacological PRS, an additional and conceptually distinct challenge arises from the limited transferability of PRS across ancestries that affects polygenic risk prediction more broadly (see the following section, “Transferability across patient populations”).

## Challenges in the translation of PRS into clinical practice

While the previous sections have outlined the potential and utility of PRS, their translation into routine clinical practice is associated with substantial challenges. In particular, these include the limited transferability of PRS across diverse populations, as well as the lack of widely adopted, harmonized guidelines for PRS development, reporting, and communication of results to patients.

### Methodological standards

A prerequisite for the clinical application of PRS is that the development, calculation, and validation of a given score can be both understood and traced in a transparent manner (for an overview of methodological foundations and PRS construction algorithms, see the review by Klinkhammer et al. in this issue). However, for many PRS generated in the research setting the respective documentation is insufficient. To improve transparency and comparability, reporting standards for PRS have been developed [17]. These include the Polygenic Risk Score Reporting Standards (PRS-RS), which provide a structured framework for the transparent reporting of PRS development and validation. These standards define the information that should be provided when describing a PRS. In particular, this information includes the reference population upon which the score is based, the methodology used in score construction, and the results of independent validation studies. The PRS-RS also recommend that researchers deposit their PRS in publicly accessible FAIR (Findable, Accessible, Interoperable, and Reusable) databases, such as the PGS Catalog (https://www.pgscatalog.org), in order to facilitate reproducibility and comparative benchmarking [18].

### Transferability across patient populations

Beyond standardized PRS generation and reporting, an equally important concern for the broad clinical implementation of PRS is ensuring equitable predictive performance across diverse populations. A weakness of many of the currently available PRS models is reduced predictive accuracy when applied to individuals whose genetic background is not adequately represented in the populations from which the underlying models were derived. Main drivers for the reduced transferability of PRS across ancestry groups are population-level differences in allele frequencies, linkage disequilibrium patterns, causal variant effect sizes, and gene-gene or gene-environment interactions [19–21]. As a result, many PRS show a substantially lower accuracy in individuals from understudied groups, e.g., persons of African, admixed, or other non-European ancestries, and even among subgroups within the same broad ancestry category [19, 22]. This limitation is also relevant within European populations, whereby subtle population structure can influence predictive performance. This challenge, which is shared by other biomarkers, is largely attributable to the significant underrepresentation of global human diversity in genome-wide association studies (GWAS), which generate the primary source data for PRS construction. To date, over 90 % of GWAS participants have been of European ancestry [23], resulting in limited score transferability to non-European ancestry groups [22, 24, 25].

In recognition of these ethical and scientific imperatives, major funding bodies have begun to take corrective action. A key initiative is the Polygenic Risk Methods in Diverse Populations (PRIMED) Consortium, which was created in 2021 by the National Human Genome Research Institute (NHGRI) and the National Cancer Institute (NCI). The two primary objectives of the PRIMED initiative are the aggregation and harmonization of existing datasets from diverse ancestry groups to support PRS development and evaluation, and the development of novel statistical methods to improve polygenic risk prediction across populations for a broad range of health and disease outcomes [26].

Despite significant methodological advances [18, 19], existing performance disparities cannot be fully resolved through computational approaches alone [27]. A critical foundation for ensuring PRS transferability, and generating accurate scores across different ancestry groups, lies in the establishment and genetic characterization of ethnically diverse and non-European biobanks. Large-scale initiatives, such as the VA Million Veteran Program [28], the All of Us Research Program [29], the China Kadoorie Biobank [30], and BioBank Japan [31], represent important steps toward establishing the diverse reference datasets needed to develop and validate PRS that perform equitably across global populations.

### Communication of PRS results and integration

While these efforts address the scientific and methodological foundations of equitable PRS development, their successful translation into clinical practice also depends on how PRS results are communicated and managed in healthcare settings. Understanding of PRS among both healthcare providers and patients remains low, and genomic risk estimates are frequently met with uncertainty, misinterpretation, or the misconception that genetic predictions are immutable [32].

Risk communication

A particular source of miscommunication is the framing of PRS results as relative risk increases, which tend to be perceived as more alarming than they may actually be in absolute terms. Expert bodies, including the International Common Disease Alliance’s (ICDA) PRS Task Force [12–15, 33] and the American College of Medical Genetics and Genomics (ACMG) [12–15, 33], recommend that PRS-derived risk estimates should be communicated as absolute risks, ideally within a defined time horizon and integrated with other established risk factors, rather than as fold-increases relative to the population average [34].

Psychosocial impact

Notably, emerging evidence suggests that the receipt of PRS results is not associated with substantial long-term negative psychosocial consequences. Rather, studies indicate potential benefits, including the promotion of positive health behaviors and encouragement of risk-appropriate screening uptake [35]. It is important to note, however, that PRS results, like other forms of disease risk information, do not, when communicated in isolation, reliably motivate lifestyle behavior changes, such as dietary modification or increased physical activity. Instead, their primary clinical benefit lies in enabling more accurate risk stratification, thereby enabling effective risk-reducing interventions, including pharmacological treatment, to be offered to those individuals who are most likely to benefit [34]. Importantly, there is also no evidence that lower- or average-risk PRS results lead to false reassurance or to a reduction in health-protective behaviors − a concern sometimes raised against the broad implementation of PRS-based risk communication [34]. These findings underscore the importance of investing in effective communication strategies and education programs to ensure that the demonstrable benefits of PRS-based risk information are not undermined by inadequate interpretation or delivery.

Clinical integration

Two essential prerequisites for the meaningful clinical integration of PRS are targeted training and continuing education of healthcare providers in the interpretation of genomic risk information, and comprehensive patient education. To this end, expert bodies have issued guidance on the appropriate use and interpretation of PRS, including the PRS Task Force of the ICDA [36] and the ACMG [37]. Both advise against the use of PRS as standalone tests, and recommend instead that they should be integrated with the consideration of established clinical risk factors, monogenic variant testing, and existing risk prediction models. Ongoing clinical pilot programs, such as the electronic MEdical Records and GEnomics (eMERGE) Network, are generating the first systematic insights into PRS implementation in routine clinical care, including the development of Genome-Informed Risk Assessment (GIRA) reports, which integrate PRS results with monogenic variant information, clinical risk parameters, and family history [38]. Similarly, large-scale national programs are investigating how PRS can be incorporated into clinical recommendations at the population level. These include the Our Future Health initiative in the UK, which is a broad population health research programme that aims to recruit up to five million volunteers in order to develop new approaches to disease prevention, detection and treatment (www.ourfuturehealth.org.uk).

### Regulatory framework: European and German perspectives

The clinical application of PRS takes place within a layered regulatory environment. At the European level, PRS-based tests offered as clinical diagnostics fall within the scope of the In Vitro Diagnostic Medical Devices Regulation (IVDR; EU 2017/746), which became fully applicable in 2022. Software for the calculation of polygenic risk scores is explicitly listed under code IVR 0402 in the MDCG guidance on IVDR codes (MDCG 2021-14), and devices for human genetic testing generally fall into risk Class C under Rule 3(j) of Annex VIII IVDR, requiring conformity assessment by a notified body. Open practical questions concern, in particular, the applicability of the health institution exemption (Article 5(5) IVDR) and the regulatory handling of PRS algorithms that are continuously updated as new GWAS data emerge.

In Germany, a further and distinct regulatory layer is provided by the Genetic Diagnostics Act (Gendiagnostikgesetz, GenDG, https://www.gesetze-im-internet.de/gendg/BJNR252900009.html), which came into force in 2010. The GenDG governs the conduct of genetic testing and the handling of genetic data in the clinical relationship, covering issues such as informed consent, genetic counseling obligations, and data protection. The Genetic Diagnostics Commission (Gendiagnostikkommission, GEKO; https://www.rki.de/DE/Institut/Organisation/Leitungsstab/GEKO/geko-node.html), which is hosted by the Robert Koch Institute, develops guidelines and recommendations for the conduct of genetic testing within this legislative framework. Clinical applications of PRS have not yet been the subject of a formal GEKO guideline or recommendation. However, the Commission’s fifth activity report, published in 2025, includes a dedicated section on the topic in Chapter IV (“Assessment of Developments in Genetic Diagnostics”): “Polygenic Risk Scores (PRS) – State of the Discussion” [39]. This summarizes international discussions concerning clinical applications of PRS, but does not comment on regulatory aspects.

For the application of PRS, the following definition in the GenDG is particularly relevant (§ 3 Nr. 7 lit. b): “a diagnostic genetic test is a genetic test aimed at determining whether genetic characteristics are present that, in conjunction with the action of certain external factors or foreign substances, may trigger a disease or health disorder.” The classification as a diagnostic rather than a predictive genetic test is important, as the latter − conceived for diseases in which a single mutation confers a high risk of a serious and potentially untreatable condition − is subject to stricter regulatory requirements for patient protection. Even where PRS can stratify the risk for severe disease, the conceptual distinction from predictive testing rests on two points: first, the genetically determined probability of falling ill is not so high as to imply a fate-like, inevitable disease course; and second, modifiable external factors, such as lifestyle or environmental exposures, substantially co-determine the actual risk and can, in principle, be addressed clinically. On this basis, PRS conceptually align with diagnostic rather than predictive genetic testing under the GenDG. In practical terms, the classification of PRS as diagnostic rather than predictive genetic testing under the GenDG means that PRS testing is subject to the medical practitioner reservation (‘Arztvorbehalt’, § 7 GenDG) and that, upon disclosure of results, genetic counseling should be offered (§ 10 Abs. 1 GenDG). However, the more stringent requirements that apply to predictive genetic testing, in particular mandatory pre- and post-test genetic counseling by appropriately qualified physicians, do not apply.

### Ethically contested applications

The expanding technical capabilities of PRS will also enable applications that must be subjected to critical scrutiny. A controversial example is PRS-based embryo selection, which is already offered commercially by companies in the United States. In this approach, PRS are used to rank embryos generated by in vitro fertilization according to predicted disease risk or other traits, going well beyond established preimplantation genetic testing for monogenic disorders. This application raises profound ethical questions regarding the instrumentalization of human life, reproductive autonomy, and societal notions of normality. PRS-based embryo selection has been critically assessed in the scientific literature both on ethical and on methodological grounds [40–42]. Methodological concerns include the limited predictive accuracy of PRS at the individual level, the complexity of gene-environment interactions underlying most common diseases, and − strikingly − the finding that embryo rankings are highly inconsistent across different PRS construction methods, such that the choice of method rather than the underlying biology may determine which embryo is selected [43]. This application is viewed with great skepticism by the medical genetics community [43].

## Outlook

Against this backdrop of both promising developments and ethically contested applications, the overall trajectory of PRS research and clinical translation remains encouraging. The coming years are likely to see an expansion of validated applications across a growing number of disease areas, driven by progress in ancestry-diverse GWAS, improved methodological standards, and the maturation of clinical implementation frameworks. The integration of PRS with other layers of genomic and clinical information − including monogenic variant data, biomarkers, and electronic health records − holds considerable promise for more precise and individualized risk assessment. PRS have the potential to make a meaningful contribution to more individualized, predictive, and preventive medicine, provided that their implementation is accompanied by rigorous evidence generation, clear clinical guidelines, and equitable access across populations.
